# Why might regional vaccinology networks fail? The case of the Dutch-Nordic Consortium

**DOI:** 10.1186/s12992-016-0176-6

**Published:** 2016-07-07

**Authors:** Jan Hendriks, Stuart Blume

**Affiliations:** Institute for Translational Vaccinology (Intravacc), Antonie van Leeuwenhoeklaan, 9, 3721 MA Bilthoven, The Netherlands; Amsterdam Institute for Social Science Research (AISSR), University of Amsterdam, P.O. Box 15718, 1001 NE Amsterdam, The Netherlands

**Keywords:** Globalization, Pneumococcal conjugate vaccine development, Regional networks, Public sector consortium, Privatization, WHO, Developing countries

## Abstract

We analyzed an attempt to develop and clinically test a pneumococcal conjugate vaccine for the developing world, undertaken by public health institutions from the Netherlands, Sweden, Denmark, Norway and Finland: the Dutch Nordic Consortium (DNC), between 1990 and 2000. Our review shows that the premature termination of the project was due less to technological and scientific challenges and more to managerial challenges and institutional policies. Various impeding events, financial and managerial challenges gradually soured the initially enthusiastic collaborative spirit until near the end the consortium struggled to complete the minimum objectives of the project. By the end of 1998, a tetravalent prototype vaccine had been made that proved safe and immunogenic in Phase 1 trials in adults and toddlers in Finland. The planned next step, to test the vaccine in Asia in infants, did not meet approval by the local authorities in Vietnam nor later in the Philippines and the project eventually stopped.

The Dutch DNC member, the National Institute of Public Health and the Environment (RIVM) learned important lessons, which subsequently were applied in a following vaccine technology transfer project, resulting in the availability at affordable prices for the developing world of a conjugate vaccine against Haemophilus influenzae type b. We conclude that vaccine development in the public domain with technology transfer as its ultimate aim requires major front-end funding, committed leadership at the highest institutional level sustained for many years and a competent recipient-manufacturer, which needs to be involved at a very early stage of the development.

At the national level, RIVM’s policy to consolidate its national manufacturing task through securing a key global health position in support of a network of public vaccine manufacturers proved insufficiently supported by the relevant ministries of the Dutch government. Difficulties to keep up with high costs, high-risk innovative vaccine development and production in a public sector setting led to the gradual loss of production tasks and to the 2009 Government decision to privatize the vaccine production tasks of the Institute.

## Background

In May 1974, the World Health Assembly adopted a resolution formally establishing what became known as the Expanded Programme on Immunization (or EPI). The principal objective was to help countries “develop or maintain immunization and surveillance programmes against some or all of the following diseases: diphtheria, pertussis, tetanus, measles, poliomyelitis, tuberculosis, smallpox and others, where applicable, according to the epidemiological situation in their respective countries”. Recognizing that this would only be possible if public health authorities had access to good quality vaccines at reasonable cost, the resolution also committed the WHO to studying the possibilities for expanding vaccine supply including “developing local competence to produce vaccines at the national level”. In the years that followed, as an increasingly globalized pharmaceutical industry increased its commitment to vaccine production, and as a consequence of the ideological shifts of the 1980s, this commitment to stimulating local vaccine production, to ‘vaccine self-sufficiency’, gradually vanished from international policy statements and resolutions.

Today there are growing signs from different regions that low and middle-income countries, concerned to ensure affordable access to vaccines for their growing populations, have a renewed interest in stimulating vaccine self-reliance. In Asia, an initiative to increase regional vaccine security started in 2014 under the auspices of the Association of South East Nations, ASEAN [1]. Also in 2014 the Organization of Islamic Countries (OIC) established a Vaccine Manufacturers Group under a program called “self-reliance in vaccine supply and production “ with a focus on the Middle East and North Africa [2]. In 2015, the African Vaccine Manufacturing Initiative (AVMI) brought together stakeholders to “develop a roadmap to reach a strategy for vaccine manufacturing and procurement in Africa”. This was followed in February 2016 by a declaration by African Ministers at a Ministerial Conference on Immunization in Africa, to increase the use of vaccines by –among other actions- promoting and investing in regional capacity for the development and production of vaccines in line with the African Union Pharmaceutical Manufacturing Plan [3]. In this paper, we analyse a previous comparable initiative, in the hope that the lessons that can be drawn from its ultimate failure will be of value to those planning these initiatives.

### Vaccine policy in the 80s

In the 80s, responding to priorities of the new ‘global health’ policy, to the availability of new sources of funds as well as more comprehensive epidemiological data, and to the efforts of the pharmaceutical industry seeking new markets, developing countries acknowledged their need for vaccines beyond the classical vaccines supplied in the context of the EPI.

In 1984 at a conference at the Rockefeller Foundation’s Bellagio Conference Centre, the Taskforce for Child Survival (TFCS) was installed to energize and transform existing international vaccine programs committed to immunizing the world’s children. Antony Robbins, director of the vaccine development and production initiative of the TFCS, initiated a more pro-active public sector role in vaccine development and strongly promoted a research incentive system called frond-end funding. Robbins and others had analyzed obstacles to development, testing, mass production and distribution of vaccines needed in developing countries and observed that whilst the UN was not equipped to produce them, manufacturers had little interest in doing so. They concluded that the impediments to develop new vaccines were chiefly of an economic and political rather than scientific nature. The classic EPI vaccines could be sold cheaply because there were no more development costs. The main obstacle for development of new vaccines with little commercial interest^1^ was that the decision to develop is left in the hands of a few institutes or commercial manufacturers in the developed world, who consider it necessary to recoup the research and development costs before selling at cost price.

Capitalizing on the new opportunities for vaccine development created by the biotechnology revolution, the TFCS subsequently developed an initiative to accelerate development of new and improved vaccines for use in developing countries through a “front-end” funding program. UNDP had suggested a revolving fund to cover cost of development. EPI buyers were to agree on long-term purchase agreements with a small surcharge to replenish these development costs over a 5 or 10 year period. The TFCS would establish such fund with a Standing Committee to manage and oversee it. WHO would select the vaccines and the TFCS would establish contracts with developers [4]. These plans for frond-end funding were presented at another Bellagio conference entitled “Protecting the World’s Children” in March 1988, which ended in the Declaration of Talloires.

The Talloire Declaration called for the global eradication of poliomyelitis by the year 2000, but also called for research and development including technology transfer on acute respiratory diseases by:

“urging national governments, multi- and bilateral development agencies, United Nation agencies, non-governmental organizations and private and voluntary groups to commit themselves to pursue research and development, including technology transfer, in support of the initiatives to control respiratory infections which hold promise in the years ahead of averting many of the 3 million childhood deaths from acute respiratory infections each year in developing countries and that are currently not prevented by immunization” [5].

After interacting with the TFCS during the 1988 Bellagio conference, public sector vaccine institutions from the Netherlands and Scandinavia responded actively to this Call for Action, which eventually led to the establishment of a ‘Dutch Nordic Consortium’ (DNC) and the pneumococcal vaccine project described below. The private industry also considered the analysis of Robbins as a step in the right direction but added that more incentives would be needed to get real commitment from industry: increased protection for another product of that company; extended patents or monopolies for certain countries, or higher prices [6]. In July 1989, the TFCS solicited specific proposals from manufacturers who wished to be considered for “front-end” assistance in developing vaccines of high value to the EPI [5]. Independent scientists reviewed proposals on vaccines against Meningococcus A/C and Meningococcus B, cholera, Japanese encephalitis and pneumococcal infections, the latter being submitted from Finland on behalf of the Finnish, Swedish and Dutch institutes. The idea was that the TFCS would commit to seek funds from its members, foundations and bilateral development programs, for one or more selected proposals. The reviewers considered the proposals on conjugate vaccines against Meningitis A/C and *S. pneumoniae* as most promising and the TFCS subsequently undertook to acquire funds [7].

### New consortia in the 90s

In September 1990, at the World Summit for Children in New York City, WHO, UNICEF, UNDP, the Rockefeller Foundation and the World Bank launched the Children’s Vaccine Initiative (CVI) as a major new global initiative to connect new technologies to advance childhood immunization. All organizations had come to realize that the manufacture of vaccines cannot be assured without taking into account the prospective development of new vaccines [8].

Around the same time, with increasingly widespread political commitment to reducing the role of the state, public sector vaccinology institutions in Europe and other regions were facing increasing challenges to their traditional responsibilities. In this context, George Siber from the Massachusetts Biological Public Health Laboratories in the US proposed the establishment of a public sector vaccine consortium. Public sector manufacturers in industrialized and developing countries could share technology for manufacturing existing vaccines and could develop orphan ”low-profit vaccines for diseases occurring mainly in developing countries or for rare or emerging diseases” [9].

In Latin America, PAHO established SIREVA in 1993 [10]. A regional system for the America’s, SIREVA supported regional initiatives among countries with vaccine production capacities (Brazil, Mexico, Cuba, Argentina, Colombia and Chile) [11]. SIREVA initiated regional pneumococcal conjugate vaccine development initially in Brazil, but this was not continued. Luciana Leite from the Butantan Institute in São Paulo, Brazil, when asked in 2011 to look back on the initiative, remembered:

“within the SIREVA consortium, Butantan started with pneumococcal vaccine development; by fermentation of the polysaccharides. Different countries were to make different polysaccharides. The conjugation technology was developed in-house from studying the literature. Butantan in São Paulo and the Oswaldo Cruz Institute in Rio de Janeiro produced the serotypes 23 and 19 respectively. Chile and Cuba joined later. The collaboration failed, because PAHO had no money: not even money to hold meetings, so people did not interact. Then SIREVA continued in terms of surveillance” (Leite, L.,2011, personal communication).

In Geneva, plans to establish a public sector consortium were also made. In January 1995, the CVI Task Force for Situation Analysis (TFSA), held a meeting on fostering partnerships on DPT and DPT based combination vaccines, where several ongoing initiatives were discussed [12]. Julie Milstien from WHO subsequently drafted a strategic background document: “Strengthening Vaccine Production: A Consortium of Public Sector Vaccine Manufacturers” [13]. The WHO had taken part in visits by the TFSA to a series of developing countries that were producing EPI vaccines predominantly in the public sector for their national immunization programs. These visits to manufacturers and the national control authorities and laboratories in those countries had identified significant gaps in quality and quantity of vaccines. The TFSA had noted that technical support to those countries had often not been effective because of “a lack of receptive management structure leading to frustration with donors and countries”. The proposed solution was a three-step process. First, countries should critically look at the cost-effectiveness and viability of vaccine production in the public sector. Second, vaccine manufacturers should develop a receptive organizational structure to be based on elements of viability of local production. Third, a coordinated system at the international level to support these processes in individual countries needed to be set up. This proposed coordinative system of a global consortium of manufacturers would be managed by WHO and would enable the sharing of management expertise and technical knowledge among its members and would ensure that international consultant advice would be consistent. It would also promote partnerships and interactions with public sector manufacturers in industrialized countries.

This global WHO plan concept referred to earlier similar proposals made by the Netherlands Institute of Public health (RIVM) [14] and the Massachusetts Public Health Laboratories in the US and it aimed to build on the ongoing SIREVA initiative. The emphasis of the proposed activities was on quality, production rationalization, regional national control laboratories, and training in Good Manufacturing Practices (GMP). The plan did not elaborate on specific work plans and research priorities [13]. However, when presented to the Fifth Annual Meeting of CVI’s Consultative Group in São Paulo in October 1995 [15], it was rejected. Despite support from several developing country producers, such as the Butantan Institute, several experts and representatives from the international vaccine industry were skeptical and expressed doubts about the viability of public sector manufacturing. Soon after, WHO silently shelved the plan. Some public sector supporting participants, such as Isaias Raw from Brazil, later expressed their opinion that the international vaccine industry saw the proposed consortium as a “cartel” (Raw I., 2011, personal communication*).* Interestingly, about 5 years later, several of the elements and proposed activities of this plan were taken on by the creation of the Developing Countries Vaccine Manufacturer’s Network (DCVMN) in 2001 [16].

### The Dutch Nordic Consortium (DNC)

**Fig. 1:**
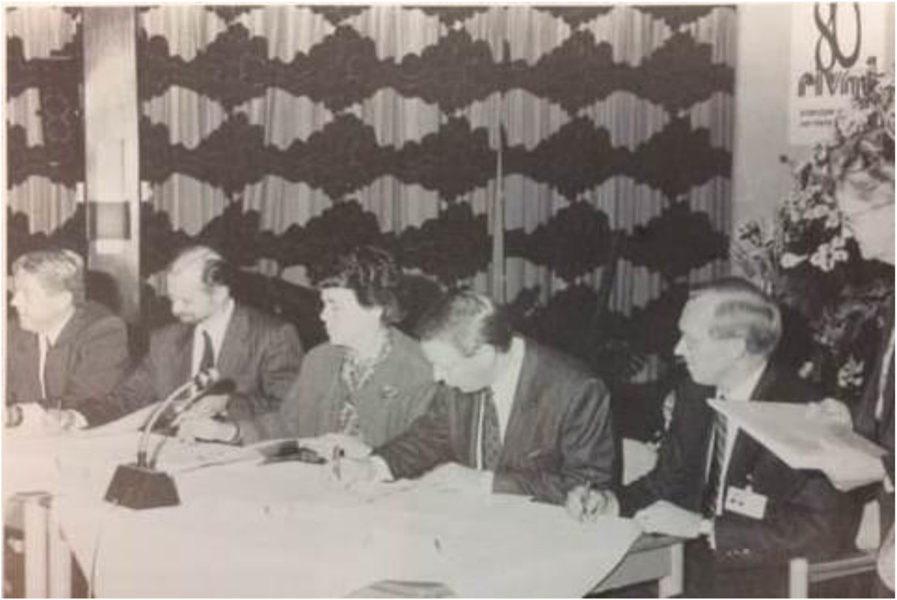
Signing of the Dutch Nordic Consortium Letter of Intent by the Directors of the Public Health Institutes in Scandinavia and the Netherlands, at the occasion of the 80^th^ year of existence of the RIVM, 25 October 1990. From left to right: B. Hareide (Norway’s Institute of Public Health), J. Huttunen (Finland’s Institute of Public Health, Mrs. R. Norberg (Sweden’s National Bacteriological Laboratory), L. Pallesen (Denmark’s Staten Serum Institute) and R. van Noort (Netherlands’ RIVM)

On 25 October 1990, RIVM celebrated its 80th year of existence with an international seminar highlighting its international cooperation in the field of health and the environment. At this seminar, health institutes from Norway, Finland, Sweden and Denmark together with the RIVM, signed a Letter of Intent to cooperate in the development of new vaccines for third world countries (Fig. 1). They decided, “to carry out projects and programs in the field of public health in the developing countries, starting with the development of a vaccine against pneumococci” [17]. Other strategic considerations also played a role. Since all Institutes were tasked by their governments to supply the national immunization programs by production or procurement it was thought that an European collaboration could reduce costs and benefit procurement in the event of any emergency [18]. This would be also advantageous for the TFCS objectives, as work would progress quicker if distributed among members. The DNC would combine the accumulated experienced expertise of the five institutes, and benefit from financial support from the TFCS, as well as from Nordic and Dutch bilateral development aid programs. Resulting products would be given to WHO and countries in the third world. Several proposals for new vaccine development were submitted to the TFCS [19].

Looking to connect the DNC plans with the recently established Children’s Vaccine Initiative (CVI), in early 1991 RIVM hosted a CVI workshop on “the Role of the Public Sector Institutions in Developing and Industrialized Countries” [20]. Participants were from UNDP, UNICEF, WHO, PAHO, the Rockefeller Foundation and included representatives from public sector manufacturers from the DNC, China, Brazil, Mexico and individual international vaccine experts. With initial funding from the Dutch Ministries of Economic Affairs and of Foreign Affairs (Development Cooperation), RIVM had initiated a Centre for training and technology transfer to establish or strengthen vaccine production facilities in a selected number of highly populated developing countries [21]. It was expected that building on the contacts and the work by the TFCS, CVI could support public health institutes in the DNC and use them to aid the public sector institutions in the developing world [22]. RIVM anticipated to become a major pillar of the CVI because, as historian William Muraskin has pointed out: “it saw its ability to help transfer vaccine technology to the third world as a major justification of RIVM’s continued existence” [22]. The objective of the workshop was to define critical issues to successful implementation of the CVI. In fact, the workshop was an effort to integrate the starting and ongoing regional initiatives (SIREVA and DNC) into CVI’s strategic plan. The workshop proceedings [20] state that participants recognized amongst others, that

“the SIREVA initiative is committed to improve the scientific and technological infrastructure and management of science for public health in Latin America; that the DNC is committed to the joint development of new vaccines and to transfer its expertise to developing countries and that regional cooperation needs to be promoted in the CVI”.

On the occasion of the First European Conference on Vaccinology, held in Annecy, March 1992, RIVM’s Director-General re-iterated his opinion on the role of public sector manufacturers in the CVI [23]. After expressing concerns about the slow development of the CVI, he proposed, referring to the DNC as an example, a more action-oriented approach with the role of the public sector in the CVI mainly in research and in providing scientific and technical support to developing countries and organizations. To do this effectively, the public sector needed to maintain a limited role with respect to vaccine production. Referring to an earlier paper by Robbins [24], he stated that:

“The new set of initiatives (such as CVI) share a recognition that public institutions must assume a central role in managing decisions about vaccine research, development, production, and distribution”.

However, when the CVI’s strategic plan was finally published in 1993, none of these proposals had been adopted. The public sector institutions were not seen as an essential part of CVI’s strategy neither individually nor acting as regional networks.

### The tetravalent pneumococcal conjugate vaccine project

The DNC embarked on the development of a conjugate vaccine project because it was thought that the pooled scientific and technical experience and expertise in the five institutions would ensure a reasonable chance of success. The Swedish and the Dutch institutes had in earlier projects [25, 26] accumulated pioneering experience in the innovative conjugation technology , and with the added capacity of the other Scandinavian institutes on serotyping, animal assays and clinical study design, the consortium basically possessed all knowledge and infrastructure to succeed. *S. pneumoniae* was chosen as a vaccine target, because of its high morbidity and mortality in developing countries.

Of the 8.8 million global annual deaths amongst children under 5 years of age in 2008, WHO estimated in 2012 that 476 000 were caused by pneumococcal infections. Disease rates and mortality are higher in developing than in industrialized settings, with the majority of deaths occurring in Africa and Asia. Although there existed a polysaccharide vaccine against the bacterium, it appeared not to be effective in children under 2 years of age. There was evidence that a conjugated vaccine, made by attaching a poorly immunogenic (polysaccharide) antigen to a carrier protein, would stimulate a more vigorous immune response and would effectively protect young children.

This choice for a, in itself rather complex,^2^ conjugation-technology approach for a vaccine against pneumococcal infections proved to be correct as shown by the subsequent emergence of a highly profitable global market for pneumococcal conjugate vaccines (PCVs) in developing countries, now shared between Pfizer and GSK [27–29].

After the signing of the DNC Letter of Intent, it still took a long time before start-up grants from the respective governments and the European Union were in place and the project could take off. Detailed bilateral collaboration agreements were made in advance to describe each partner’s specific contributions and responsibilities in proportion to the financial reimbursements from received grants.

Initially a vaccine was envisaged with the four serotypes that were globally the most frequent cause of pneumococcal disease in children under 2 years of age (6B, 14, 19 F and 23 F). When successful, another four serotypes, with specificity for developing countries would be added to make it an eight-valent vaccine.

The laboratory scale development of saccharide-protein conjugates started at the RIVM in the Netherlands and at the Swedish Bacteriological Laboratory (SBL) in the second half of 1993. RIVM and SBL had both independently developed different conjugation technologies.^3^ The DNC management committee decided to use the Swedish technology for the DNC vaccine and chose tetanus toxoid from RIVM as the carrier protein. Norway would apply an animal model to test experimental conjugate vaccines. The SSI in Denmark, that housed the WHO Reference Laboratory on pneumococcal isolates and typing, was to develop assays to measure the immune response in animals against each of the specific pneumococcal serotypes that were to be in the DNC vaccine. Finland’s National Institute would take responsibility for the design and operational activities needed for the clinical studies.

**Fig. 2:**
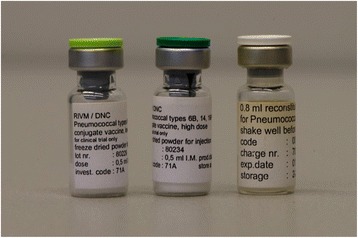
The DNC tetra-valent pneumococcal conjugate vaccine made in 1998. The left and middle single-dose glass vials contain respectively a low-dose (LD) and a high-dose (3xLD) freeze-dried formulation with the four serotypes 6B, 14, 19 F and 23 F all conjugated individually to tetanus toxoid. The vial on the right contains the reconstitution fluid in which the vaccine dose is dissolved just prior to the vaccination

In the course of the project, which lasted about 10 years, various impeding events and managerial challenges, described in detail below, then gradually soured the initially enthusiastic spirit of collaboration . By the end of the decade the parties that had remained in the consortium had to struggle to complete the minimum objectives agreed with the European Commission, which by then remained as the only financial sponsor. Despite these and other obstacles, by late 1998 the DNC managed to produce a prototype vaccine that was ready for field-testing in developing countries (Fig. 2). This prototype proved safe and immunogenic in animals. Two subsequent small-scale Phase 1 clinical studies in adults and toddlers in Finland confirmed its safety and indicated immunogenicity [30, 31]. The planned next step was to test the vaccine in Asia in infants. Unfortunately, it was not approved by the local authorities in Vietnam nor later in the Philippines and the project had eventually to be abandoned.

### The challenges that faced the DNC

#### Financial and managerial constraints

Funding was a major challenge throughout the project, as it had to be obtained from different sources each with tedious and time-consuming application procedures. In addition to the Institutes’ own financial contribution, the DNC managed to obtain grants from the European Commission, the Netherlands Ministry of Development Cooperation and the Scandinavian Development Agencies for the development, testing and its production at RIVM. The European Commission initially contributed with a grant for a 5 year period (1993–1997) for the vaccine development. This was followed by a second 5 year grant (1997- April 2002), later extended to October 2003. The second EU grant served to clinically test the DNC vaccine in Finland and Vietnam and formed part of another EU funded project (ARIVAC2), coordinated by the Finnish DNC Partner, which was to test an eleven-valent pneumococcal conjugate vaccine developed by Pasteur Merieux Vaccines (now Sanofi Pasteur) in the Philippines.

Overall, the DNC program suffered from too little and insufficient upfront funding that had to be collected from different sources, requiring substantial energy and time of investigators. Despite the long-term preparations and hopeful early indications, in the end, neither the TFCS nor the CVI contributed any funds at all. The modest funds that were raised eventually came with many administrative restrictions, making it hard to proceed fast. Moreover, the EU was not interested in financing the collaboration with SIREVA, that the DNC hoped for.

#### Privatization and patent claims

In 1993, the Swedish Government privatized the vaccine production of the SBL, establishing a new entity (SBL-Vaccin) and the Swedish Institute for Infectious Disease Control (SIIDC), which included the research and epidemiological surveillance tasks of the previous SBL. The responsibilities of the Swedish partner in the DNC were taken over by the SIIDC. This major organizational chance within one of the partners seriously affected the institutional collaboration. Added to this, in 1994 some controversy arose between RIVM and SIIDC in 1994 regarding a patent application filed independently by SIIDC on the Swedish conjugation technology, selected for use in the DNC vaccine. The scientific paper describing the technology, published in 2000, expressed the hope that the application of the “Swedish” conjugation technology would reduce vaccine costs for developing countries:

“Taking into account their simplicity and feasibility for large-scale preparation of pneumococcal polysaccharide conjugate vaccines at costs appropriate for the general use in developing countries, we hope that the described techniques will be further exploited” [32].

In return, RIVM also decided to independently file a patent application on its own technology with a plan to provide later on exclusive licenses to partners in developing countries.

In retrospect, these cases of unilateral patent applications had a negative impact on the working relations within the consortium.

#### The project’s leader moves to the private sector

In the second half of 1996, the key scientist and informal leader of the DNC pneumococcal vaccine project, Jan Poolman decided to join the private sector. Heading the RIVM laboratory for bacterial vaccine development for 10 years, he had become increasingly frustated in his ambition to drive development of bacterial vaccines forward in an institutional climate that at times seemed to restrict instead of facilitate progress. Poolman had repeatedly argued for more capacity and investments in development programs targeting the introduction of new meningococcal and pneumococcal vaccines into the Netherlands Immunization Program. Conflicts with other units and projects in the vaccine division on competing for access to essential experienced technical staff and specialized laboratory suites for vaccine production emerged. In addition to its national tasks, the Institute was at that time struggling with several international vaccine projects, including a large EPI vaccine technology transfer project with the Chinese Government funded by a soft loan from the World Bank [33]. Pressures were such that the Institute’s management offered little support to Poolman’s development projects. As a national institute under the Netherlands Ministry of Health, convincing decision makers at the Ministry to increase budgets for costly vaccine development and clinical testing, with no promise of measurable short-term returns was becoming increasingly difficult. Poolman grew increasingly disappointed and in 1996 accepted an offer to join GSK in Belgium, where he later became one of the co-developers of GSK’s ten-valent pneumococcal conjugate vaccines PVC10 (Synflorix) and several new vaccines against pertussis, *Haemophilus influenza* type b (Hib) and meningococcus. His conclusion at that time was that

“vaccine development and production is not any longer possible in the public sector due to inadequate resources, lack of infrastructure and too little will to make it a success” (Poolman, J 1997, personal communication).

The DNC was left without a leader during the transition period that followed. Perhaps coincidentally, at around the same time, another advocate of collaborative public sector vaccine development, George Siber from the Massachusetts Public Health Biologicals Laboratory, had come to a similar conclusion and moved as well to private industry. He jointed Wyeth-Lederle in 1996 where he subsequently played a role in the development and commercialization of the world’s first licensed pneumococcal conjugate vaccine, the seven-valent PCV7 (Prevnar).

#### Vietnam and the Philippines withhold approval for immunogenicity studies in infants

Around mid-1995, DNC members began to consider the region or country where the vaccine could be tested in the field. SIREVA/PAHO with its network in Latin America seemed a logic choice [34]. Bilaterally, RIVM also had good contacts with PAHO on training programs about vaccine quality control and quality assurance. It also had contacts with vaccine manufacturers in Latin America, such as the Butantan Institute, also involved in SIREVA.

The DNC management proposed to the European Commission to field-test the DNC vaccine in the Latin American region, by first building up local capacity for pneumococcal quality control and analytical tests followed by vaccine production technology transfer, for example to the Butantan Institute. However, this did not fit the European Commission policy at that time. The relevant EU working program had prioritized the Asian region over the Latin America region. As a result there were no funds for a SIREVA-DNC collaboration [35]. On top of this, the Butantan management appeared not to be interested in facilitating a field test in Brazil with a DNC vaccine if this vaccine had not been produced in Brazil first. Thus, for its second grant proposal to the EU, the DNC turned its attention to Asia and in Vietnam. The Danish DNC member, SSI, had good contacts there through the Academic Hospital in Copenhagen and a large paediatric hospital in Ho Chi Minh City.

With the second EU grant in place, sites for the clinical studies in infants were prepared in southern Vietnam and Vietnamese staff was sent for training to Denmark, where the serological analysis was to be done. The Vietnamese study investigators submitted a formal application to the regulatory authorities in Hanoi. The application included a protocol for clinical trials and a brochure for clinical investigators, made by the DNC and proposed a field study in southern Vietnam with the tetravalent DNC pneumococcal conjugate vaccine produced by RIVM. However, the regulatory body’s ethical review committee withheld approval. Unaccustomed to authorizing new investigational vaccines not yet licensed in other countries and that had not been produced in Vietnam, their main concern was with the possibility of serious adverse events. This was despite the documented evidence of successful safety testing in Finnish Phase 1 studies. At the time of application, rumors were circulating on adverse events caused by locally made vaccines and the regulatory body did not want to take any risks. [36, 37]. This rejection was a major setback and it necessitated a search for a new study in another country.

By the end of 2002, the consortium management agreed with the European Commission to transfer the site of the Phase 2 trial in infants from Vietnam to the Philippines. By this time RIVM had announced that due to other priorities it would not to continue the clinical development of the vaccine. Nevertheless, the reasoning was that, if the vaccine proved immunogenic, the prototype could be offered for further development by another manufacturer, possibly in a developing country. However, the ethical review board of the partner institute in the Philippines, the RITM, also witheld its approval. Since RIVM would not continue the clinical development, the availability of the vaccine was uncertain. In view of this, the risks of subjecting these infants to an experimental vaccine outweighed its uncertain benefits [38] . This second refusal marked the end of the DNC pneumococcal vaccine development project.

The decision by the Vietnamese regulatory body is best understood as due to its weak regulatory capacity. At that time it had no procedures for dealing with applications for investigational new vaccines not made in Vietnam. In fact, the weakness of the Vietnamese national regulatory authority has been a concern for many decades, and very recently, after an intensive capacity building program, has it been approved as the Vietnamese NRA has reached the international status of being fully competent to exercise the six essential regulatory functions seen as essential by WHO [39]. The rejection by the ethical review in the Philippines was due in part to the information that RIVM would not continue the vaccine development. It also reflected the availability of a commercial seven-valent vaccine that covered all the serotypes (and more) that were contained in the DNC vaccine. The only justification for continuation, that the technology might be transferred in the future to manufacturers in developing countries, proved not to be a convincing argument. None such manufacturer had been identified.

### What can we learn from this experience?

#### The consortium was not endorsed by the global vaccine community

Despite the initially active interactions between the DNC and the TFCS, and later the CVI, eventually neither concrete collaboration nor front-ending funding materialized from that side. The CVI, once established, became the international forum for UN organizations, policymakers, technical agencies, academia and industry to discuss all matters regarding vaccine development and vaccine supply for developing countries. The DNC was unable to become incorporated into the CVI, one of the reasons being that the Netherlands and the Scandinavian countries, as an important donor countries to WHO’s EPI program expressed concerns that the “US-driven” research driven and technology-focused CVI movement would jeopardize EPI country delivery programs [22]. On several occasions, the RIVM management, emphasizing its international technology transfer experience, reflected in a 1995 RIVM International Vaccine Policy Brochure [14], called for an action-oriented approach and promoted a stronger role of public sector vaccine manufacturing in the CVI, but the impact remained minimal. Over time, the CVI moved gradually towards more partnerships with the international vaccine industry.

#### The lack of a recipient vaccine manufacturer proved a major weakness

The attempt to establish a “bridging”” relationship between SIREVA/PAHO and the DNC did not materialize. In retrospect, we argue that it was a major weakness that no developing country partner had been engaged from the early stages onwards. By not including such partner from the beginning, the research-focused consortium was insufficiently able to manage the project from the perspective of either a manufacturing or a regulatory recipient. Little attention was paid to process-upscaling or to preparing a technology transfer package to engage to the Butantan Institute in Brazil. There had been no early interactions with regulatory authorities in Vietnam and the Philippines in anticipation of the clinical studies.

#### Privatization policies hindered public sector collaboration

Over time, the Institutes’ senior management had less and less interest to the pneumococcal vaccine development project. The plan to hold regular meetings of the DNC Board of Director was never implemented. Due to ever-continuing institutional reorganizations and shifts in institutional vaccine development priorities,^4^ the research-based consortium team worked in an environment that lacked a real sense of urgency. The Boards of the respective Institutes turned out not to remain fully committed to the pneumococcal vaccine project”. Peter Bootsma, who co-managed the DNC project from the side of RIVM, remembered in 2015:

“When it suited politically the “Dutch Nordic Consortium” concept was enthusiastically exploited, but real commitment backed with substantial funding, that was an entirely different matter” (Bootsma P.,2015, personal communication).

The privatization of vaccine production has since continued among all the Institutes that formed the DNC. The small populations of each of the DNC countries makes vaccine manufacturing economically unsustainable. Sweden stopped production in the public domain in 1993 (Olin, P., 1998, personal communication) . Finland stopped in 2003 [40]; the Netherlands in 2009 [41], In December 2014, Denmark has initiated the privatization of its vaccine production activities [42]. Norway still maintains a contract development and manufacturing organization to serve biotechnology companies, but this facility will be shut down in 2017 (H. Nøkleby, 2016, personal communication). At the Board level of the Institutes, the initial need to guard their interests through a regional consortium mechanism started to decline almost as soon as it was expressed. As early as 1996 Lars Pallisen, Director of the Danish SSI, told European vaccine manufacturers:

”The DNC was useful in the beginning because at that time some countries were not yet members of the EU; but it was not so successful with the exception of R&D pneumococcal vaccine development” [43].

Since meanwhile all DNC countries have now stopped vaccine manufacturing in the public domain, DNC collaboration on vaccine issues has ceased to exist at the policy level. The one common interest in vaccines that has remained is the sharing of best practices on vaccine purchases from industry for their respective national immunization programs of vaccines. Increasingly, such common interests are guarded through mechanisms of joint procurement through the European Union [44].

## Concluding remarks

In conclusion, collaborative vaccine development on common political grounds, but with insufficient upfront funding and unclear end-goals is a risky undertaking and unlikely to succeed. Although a promising tetravalent pneumococcal prototype vaccine resulted from this effort, it was not taken further due to a variety of policy-related factors described in this case study.

The sobering Dutch Nordic Consortium experience formed the basis within RIVM to design, towards the end of the 90s, a less ambitious and technically simpler development and technology transfer project for a monovalent conjugate vaccine against *Haemophilus influenza* type b (cHib). The key difference with the DNC program was that this time the management approach was entirely partner- and regulatory driven. The goal of the cHib project was straightforward: transfer of technology for an already licensed vaccine, making it a “me too” product and therefore easier for regulators. Most importantly, the early involvement of future recipient manufacturing partners (who co-financed the research and development) ensured that every decision to be taken was evaluated from a receiving partner’s perspective as well as from a regulatory perspective and possible impact on the time to license [45, 46].

The profile of the RIVM as an advanced European public sector vaccine development and manufacturing institute actively sharing technology with developing countries has faded over the last decades. Despite some notable successes in international vaccine technology capacity building and transfer [33] and the cHib project, the national mission to develop and produce new vaccines for the Dutch national immunization program, became politically unsustainable. The Institute’s policy to consolidate its national manufacturing task through securing a key global health position in support of a network of public vaccine manufacturers [14] found insufficient support from the Dutch government, nor from WHO, despite a strong appeal in 1999 by the Dutch Minister of Health for a core-membership in the GAVI Board for RIVM’s Director-General [47]. Difficulties to keep up with high costs, high-risk innovative vaccine development and production in a public sector setting led to the gradual loss of production tasks and to the 2009 decision by the Government to privatize the vaccine production tasks of the Institute.

## Abbreviations

AMC, advanced market commitment; cHib, conjugate *Haemophilus influenzae* type b vaccine; CVI, Children’s Vaccine Initiative; DNC, Dutch Nordic Consortium; EPI, Expanded Programme on Immunization; GAVI, Global Alliance for Vaccines and Immunization; GMP, good manufacturing practice; MSF, Médicins Sans Frontières; PAHO, Pan American Health Organization; PCV, Pneumococcal conjugate vaccine; RITM, Research Institute of Tropical Medicine; RIVM, National Institute of Public Health and the Environment, the Netherlands; SBL, State Bacteriological Laboratory, Sweden; SIIDC, Swedish Institute for Infectious Diseases, Sweden; SIREVA, SIstema de REdes de Vigilancia de los Agentes bacterianos responsables de neumonía y meningitis; SSI, Staten Serum Institute, Denmark; TFCS, taskforce for child survival; UNDP, United Nations Development Programme

## Footnotes

1 At that time, a new conjugate vaccine against pneumococcal infections was regarded to be of little commercial interest.

2 Jan Poolman, who left RIVM in 1996 to join GSK, co-authored a review on the history of pneumococcal conjugate vaccine development in 2013. That paper describes the strategic importance of choosing the right combination of technical factors that illustrate the complexity of manufacturing of polyvalent conjugate vaccines, such as establishing the correct dose, the best carrier-protein for each of the serotypes and the most appropriate conjugation technology [48].

3 The Swedish and Dutch conjugation technologies employed in the DNC project differ in the way how the polysaccharide is linked to the carrier protein. The Swedish method used thiolation of polysaccharides with 2-iminothiolane [32, 49], followed by coupling to the bromoacetylated carrier protein to obtain thioether-linked PS-protein conjugates. The advantages are that it is fast and suitable for larger polysaccharides and causes less cross-linking. The RIVM method used reductive amination of polysaccharides with cystamine to obtain thiolated polysaccharides [50].

4 For example: a dual strategy for pneumococcal vaccines (national/international) within RIVM, led to a lack of focus and delays: while for developing countries a conjugation strategy was followed in the DNC project, RIVM had simultaneously started research for the Dutch immunization program on a vaccine development approach based on a common pneumococcal protein, “pneumolysin” to which it had acquired a patent. In 1997, the RIVM management expected that the Wyeth Lederle PCV7 vaccine would reach the Dutch market within 2 or 3 years. Negotiations with Lederle were considered to exchange RIVM’s pneumolysoid patent and know-how for access to Lederle’s conjugate product [51].
